# Patient Age Predicts Nasal Septal Deviation in Orthognathic Surgery: A Non-Randomized Clinical Trial of 102 Participants

**DOI:** 10.3390/medicina60122000

**Published:** 2024-12-03

**Authors:** Juergen Taxis, Henrik-Robert Florian, Gerardo Napodano, Maximilian Rink, Felix Nieberle, Katja Himmelstoß, Sophia R. Lindner, Tobias Ettl, Torsten E. Reichert, Waltraud Waiss

**Affiliations:** 1 Department of Cranio- and Maxillofacial Surgery, University Hospital Regensburg, Franz-Josef-Strauß-Allee 11, 93053 Regensburg, Germany; henrik-robert.florian@stud.uni-regensburg.de (H.-R.F.); felix.nieberle@ukr.de (F.N.); katja.himmelstoss@ukr.de (K.H.); sophia-rebecca.lindner@ukr.de (S.R.L.); tobias.ettl@ukr.de (T.E.); torsten.reichert@ukr.de (T.E.R.); waltraud.waiss@ukr.de (W.W.); 2 Department of Radiology, University Hospital Regensburg, Franz-Josef-Strauß-Allee 11, 93053 Regensburg, Germany; gerardo.napodano@ukr.de; 3 Department of Otorhinolaryngology, Head and Neck Surgery, University Hospital Regensburg, Franz-Josef-Strauß-Allee 11, 93053 Regensburg, Germany; maximilian.rink@ukr.de

**Keywords:** orthognathic surgery, Le Fort I osteotomy, nasal septal deviation, nasal septum, CBCT

## Abstract

*Background and Objectives*: Orthognathic surgery is used to restore a correct anatomical and functional relationship between the jaws, with postoperative nasal septal deviation (NSD) being a common complication of Le Fort I osteotomy (LF-IO). The aim of this study was to evaluate the occurrence of NSD after LF-IO and to identify possible risk factors. *Materials and Methods*: Pre- and postoperative cone beam computed tomography (CBCT) scans from 2018 to 2023 of 102 patients after LF-IO were analyzed. After categorizing the preoperative NSDs according to the Mladina classification, the next step was to measure the angle of deviation and classify the severity grades. Pre- and postoperative NSDs were compared using a paired Wilcoxon signed-rank test and postoperative changes in NSD were correlated with surgery-relevant characteristics by calculating Spearman’s correlation coefficients. *Results*: Postoperatively, an increase in NSD was observed in 62 cases and 35 patients showed a decrease. In both cases with an increase and a decrease in NSD, the preoperatively measured deviations showed a highly significant difference compared to postoperative NSDs (both *p* < 0.001). Age correlated significantly with increases in deviation (*r* = 0.28, *p* = 0.014, CI: −1.0–−0.068) and anterior maxillary displacement showed a significant correlation with a decrease in NSD (*r* = 0.296, *p* = 0.042, CI: 0.006–1.0). Gender, cranial and caudal movements of the maxilla had no influence on the results of the NSDs. *Conclusions*: LF-IO has an influence on NSD and can both intensify and attenuate it. In addition, the risk of an increase in nasal deviation after this surgical procedure rises with the patient’s age and decreases with anterior displacement of the maxilla.

## 1. Introduction

Orthognathic surgery is used to restore a correct anatomical and functional relationship between the affected structures in facial skeletal anomalies [1,2,3]. This surgical therapy can be performed as an isolated or combined osteotomy of the maxilla or mandible [3]. Osteotomy of the maxilla in the sense of a Le Fort I osteotomy (LF-IO) with the aim of correcting a dentofacial anomaly was first performed by M. Wassmund in 1921 [4,5]. Accordingly, the Le Fort I plane runs posteriorly along the tooth roots and includes the pterygomaxillary connection [4]. This surgical technique allows the maxilla to be displaced in a transverse, vertical and sagittal direction and thus enables the treatment of skeletal class II and III dysgnathia [1,3,4,6]. After detachment from the maxilla, successful downfracture, displacement and osteosynthetic fixation of the maxilla in the new position, the nasal septum is refixed to the anterior nasal spine with a suture [2,4,7].

Due to the anatomical positional relationship, surgical repositioning of the maxilla can cause a nasal septal deviation (NSD) in addition to an increase in the nasolabial angle and nasal obstruction [1,8,9]. Favored by an insufficient reduction in the cartilaginous part of the septum, NSDs not only have aesthetic consequences, such as deviation of the nasal tip, but also functional complaints, such as restricted nasal breathing [1,10,11,12,13]. Consequently, this can significantly impair the overall quality of life and necessitate subsequent surgical correction of the septum [14,15].

In previous studies, the occurrence of an NSD has been reported as a common complication of LF-IO [13,16,17,18]. On the one hand, the increase in NSD due to cranial and anterior displacement of the maxilla has been described [1,17]. On the other hand, Moroi et al. and Baeg et al. found no influence of LF-IO on NSD and concluded that different methods of maxillary displacement did not influence NSD [19,20]. Similarly, Atakan et al. were unable to demonstrate a significant NSD after impaction of the maxilla with LF-IO [21]. Overall, we found only a few studies with a small number of cases that focused on the factors influencing NSD after LF-IO. However, we believe that the identification of such risk factors could help in the planning of orthognathic surgery cases and thus contribute to minimizing septal complications and improving clinical outcomes.

The aim of this study was to analyze the incidence of nasal septal deviation after Le Fort I osteotomy by evaluating cone beam computed tomography (CBCT) images. In addition, possible factors that could influence the occurrence of septal deviation were evaluated. The null hypothesis in this context was that patient age has no influence on the postoperative occurrence of NSD.

## 2. Materials and Methods

### 2.1. Patient Selection and Data Collection

This study examined the cases of patients who underwent a Le Fort I osteotomy at the Department of Cranio- and Maxillofacial Surgery at the University Hospital Regensburg between 2018 and 2023. The inclusion criteria for this study required the availability of pre- and postoperative CBCT images taken at least six months apart and complete imaging of the nasal septum on the radiological images. Also included were precise values regarding the displacement of the maxilla. Furthermore, only patients who had not previously undergone this type of procedure and had no history of cleft jaw and palate or the presence of syndromes were considered. Since patients with such a medical history could potentially have an altered tissue structure in the area of the nasal septum due to previous surgical procedures, these exclusion criteria were chosen to reduce interfering factors. Of course, this limited the generalizability of the results, as only a subset of the population could be represented. Further information such as the patient’s age at the time of surgery, the indication for the procedure and the extent of the individual displacements was collected. A total of 102 patients could thus be included in the study. All procedures were in accordance with the Declaration of Helsinki. Based on the retrospective analysis and a fully anonymized set of clinical data, and in accordance with the Ethics Committee’s decision (Approval No. 24-3689-104), signing informed consent was not required. Informed consent was obtained from the patients for the use of the intraoperative patient photos in this work.

### 2.2. Surgical Procedure

**Figure 1:**
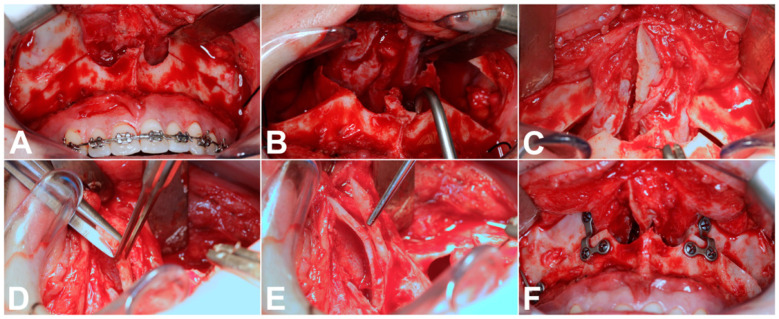
Displacement of maxilla with LF-IO (**A**), downfracture (**B**), exposure of nasal septum (**C**), reduction in nasal septum (**D**), reduced nasal septum with resected part in forceps (**E**) and fixation of maxilla in new position with 4 L-shaped titanium plates (**F**).

After nasotracheal intubation, a circular incision was performed vestibular and cranial to the mucogingival junction from the central incisor to the first molars, including the mucosa, muscles and periosteum. Following exposure of the piriform aperture, the zygomaticoalveolar crest on both sides and detachment of the nasal tubes from the bony nasal floor, we performed a conventional LF-IO with an oscillating saw in the area of the zygomaticoalveolar crest, in the facial maxillary sinus wall and in the area of the lateral nasal wall. The nasal septum was then separated from the part of the maxilla to be mobilized using a nasal septum osteotome. We then checked and completed the continuous separation of the lateral nasal wall with a chisel. We next detached the maxilla from the pterygoid process using the pterygoid osteotome, followed by the downfracture with the bone hook and a bidigital counter palpation in the nasal root area. We first performed an ostectomy of the remaining parts of the nasal crest using a bone rongeur and a rotary instrument. The nasal septum was then exposed and, particularly in the case of planned impactions of the maxilla, we shortened and straightened parts of the bony and cartilaginous nasal septum as standard using scissors and a large bone bur. This was followed by the insertion of a surgical splint. After interference between the inferior part of the nasal septum and the nasal floor was excluded, the movable maxilla could be fixed in the target position with 4 L-shaped titanium plates and screws paranasal and in the area of the zygomaticoalveolar crest. Furthermore, the nasal septum was refixed centrally via a drill hole on the anterior nasal spine before the paranasal muscles were readapted and the gingiva closed. Figure 1 shows the different surgical steps.

### 2.3. Radiological Analysis

The radiological analysis was based on CBCT images obtained with a Planmeca Viso G7 (Planmeca Oy, Helsinki, Finland, preoperative 100 kV, 50 mAs, 601 mGycm^2^, postoperative 100 kV, 12.5 mAs, 152 mGycm^2^) and imported into RadiAnt DICOM Viewer 2020.2.3 (Medixant, Poznań, Poland) for further analysis.

### 2.4. Mladina Classification

In the first step, the NSDs in the preoperative CBCT were categorized according to the Mladina classification in order to obtain an overview of the initial situation. The Mladina classification was published in 1987 by Mladina et al. and comprises seven types, including the description of vertical (type 1–4) and horizontal (type 5–6) deviations of the nasal septum, while type 7 is formed from mixed forms of the individual types [22,23,24]. Cases without NSD were described as class 0. Two investigators (J.T. and H.-R.F.) independently assigned the patient cases to the respective groups, then checked for agreement in the form of consensus and finally classified them in order to avoid possible misclassifications.

### 2.5. Determination of NSD

**Figure 2:**
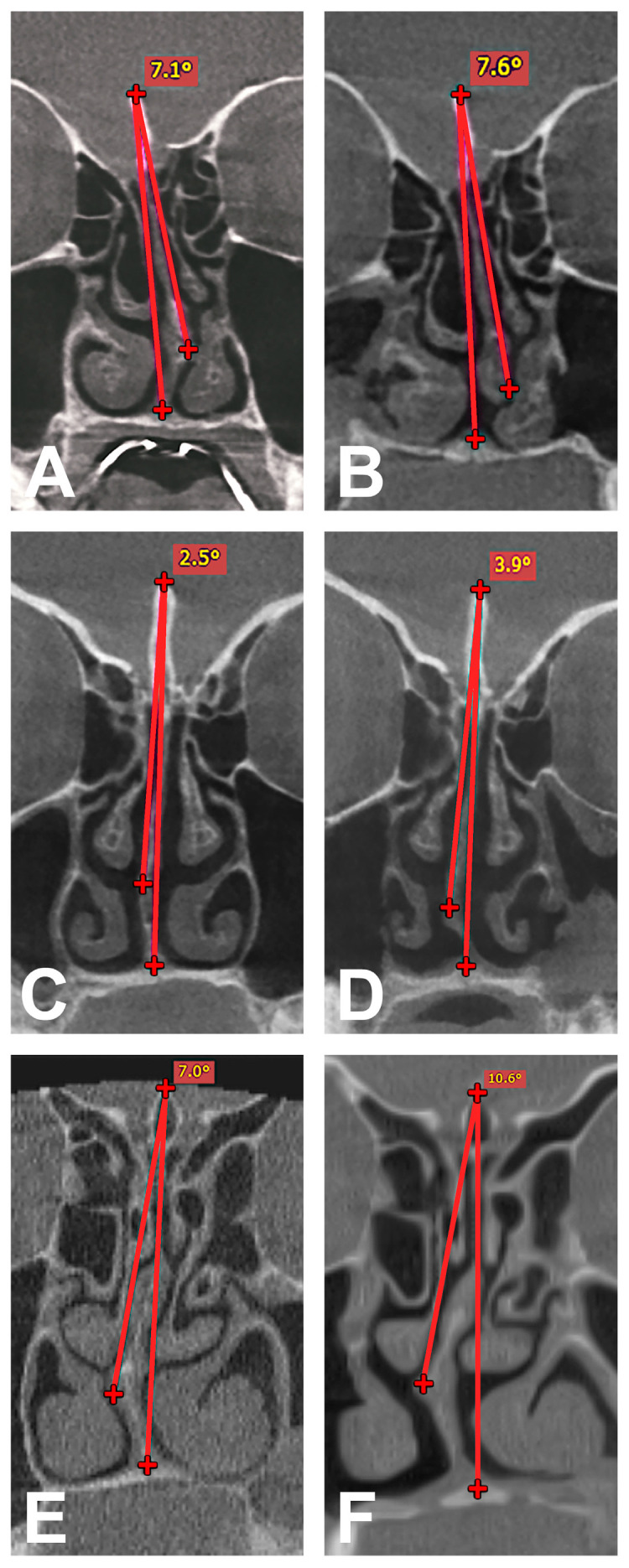
Angle measurement with increase in NSD in coronal view between two constructed lines (red line 1 between highest point of crista galli and most lateral structure at the transition from cartilaginous to bony nasal septum; red line 2 between highest point of crista galli and lower end of septum) and classification into grades. Pre- (**A**) and postoperative measurement (**B**) with grade I increase, pre- (**C**) and postoperative measurement (**D**) with grade II increase and pre- (**E**) and postoperative measurement (**F**) with grade III increase.

**Figure 3:**
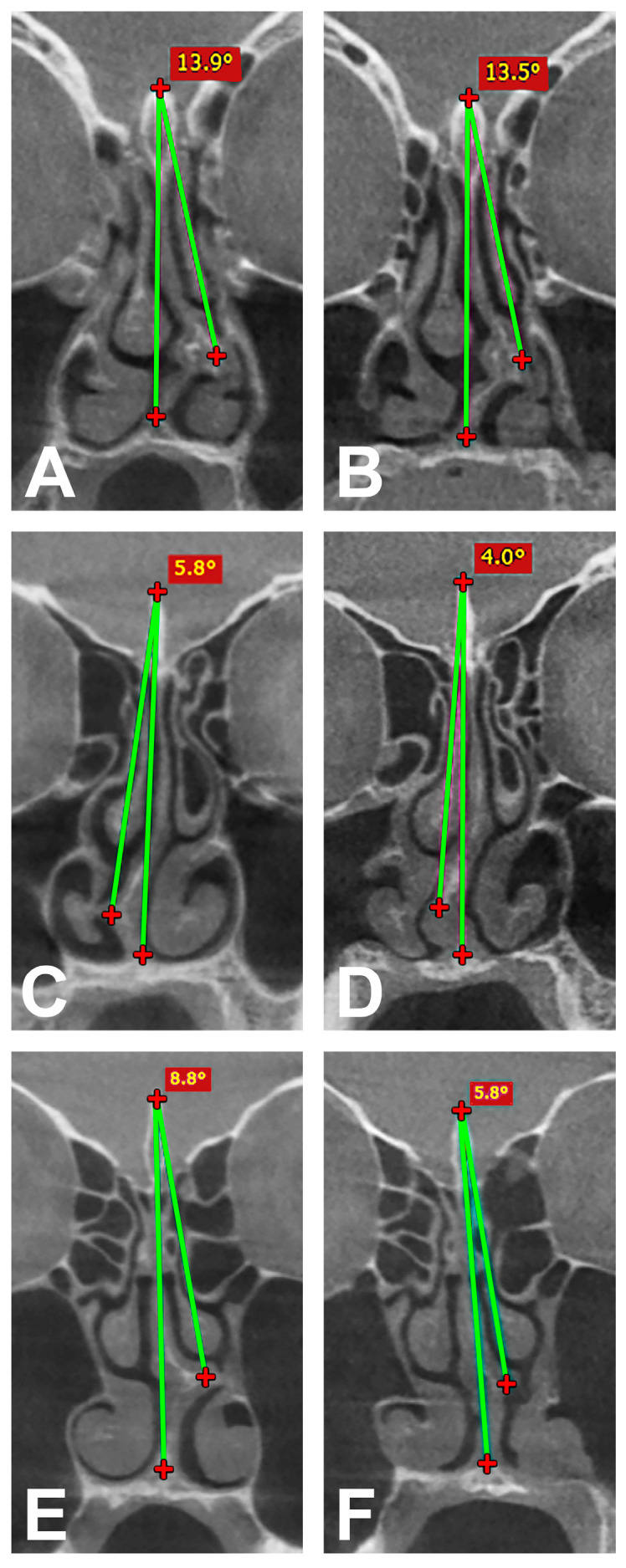
Angle measurement with decrease in NSD in coronal view between two constructed lines (green line 1 between highest point of crista galli and most lateral structure at the transition from cartilaginous to bony nasal septum; green line 2 between highest point of crista galli and lower end of septum) and classified into grades. Pre- (**A**) and postoperative measurement (**B**) with grade I decrease, pre- (**C**) and postoperative measurement (**D**) with grade II decrease and pre- (**E**) and postoperative measurement (**F**) with grade III decrease.

The NSD was then measured linearly according to the procedure of Rattana-Arpha et al. [13]. An angle was constructed in the coronal view of the preoperative CBCT images for this purpose. Initially, the highest point of the crista galli was connected to the most lateral structure at the transition from cartilaginous to bony nasal septum by the first line. A second line was then drawn from the previous point of the crista galli to the lower end of the septum at the transition to the maxilla and finally the angle between the two lines described was measured at the crista galli. The postoperative CBCT images were also measured in the same way. Both the pre- and postoperative measurements were carried out again by two investigators (J.T. and H.-R.F.) for each patient in order to minimize measurement errors. In the next step, a mean value for the pre- and postoperative deviation was calculated from the values obtained and the change in deviation was then determined. A grading system consisting of four degrees of severity each for the decrease and increase in NSD was then created and the values distributed accordingly. The value range for severity grade I extended from 0° to +0.5° for the increase and from 0° to −0.5° for the decrease in NSD. The further gradation for grade II increase was +0.5°–+3.0°, for grade III it was +3.0°–+6.0° and for grade IV, the values were > +6.0°. The same gradation was used for the decrease in NSD for grade II to IV, whereby the values were correspondingly negative. The different grades of increase and decrease in NSD in the analyzed cases are shown in Figure 2 and Figure 3.

### 2.6. Statistical Analysis

The statistical analysis was performed with IBM SPSS Statistics 29.0 (IBM Corp., Armonk, NY, USA). The median, mean and standard deviation (SD) as well as the minimum and maximum pre- and postoperative deviation measurements were calculated. A paired Wilcoxon signed-rank test was used to compare the differences between pre- and postoperative NDS. After dividing the deviations into subgroups, bivariate Spearman correlation analyses were performed between the increase or decrease in NSD and the respective variables. Additionally, we computed 95% confidence intervals (CIs) and the significance level was defined as *p* < 0.05.

## 3. Results

### 3.1. Characterization of the Patient Cohort

During the observation period of 6 years, 102 patients met the previously mentioned inclusion criteria. A total of 60 patients (58.8%) were female and 42 patients (41.2%) were male, with an average age of 28.12 ± 9.41 years (range: 17 to 56 years). Of these, 50 patients (49%) had class II dysgnathia and 47 patients (46%) had class III dysgnathia. Circular open bite was present in 2 patients (2%) and anterior open bite, mandibular retrognathism or prognathism in one patient each (1%). Looking at the isolated movements of the maxilla, cranial repositioning occurred in 36 patients and anterior displacement in 96 cases. The maxilla was moved caudally in 18 patients and no movement took place posteriorly. After classification of the initial NSD according to the Mladina classification, class 5 had the most cases with 29 patients (28.4%), followed by class 3 with 19 cases (18.6%) and class 6 with 13 cases (12.8%). Class 4 had 12 patients (11.8%), class 2 and 7 each had 11 patients (10.8%) and class 0 had 4 patients (3.9%). Class 1 was represented by 3 patients (2.9%). Increases in NSD with grade I severity were observed in 33 patients (32.2%) and with grade II in 27 patients (26.5%). A grade III increase in NSD was present in 2 patients (2%). In contrast, a grade I decrease in NSD was observed in 18 cases (17.6%) and a grade II decrease in 15 cases (14.7%). Two patients (2%) showed a grade III decrease and in five patients (4.9%) the septum was unchanged postoperatively. No grade IV increase or decrease in deviation was observed in any case. The characteristics of the patient cohort are listed in Table 1.

### 3.2. Comparison of Pre- and Postoperative Changes in NSD

**Figure 4:**
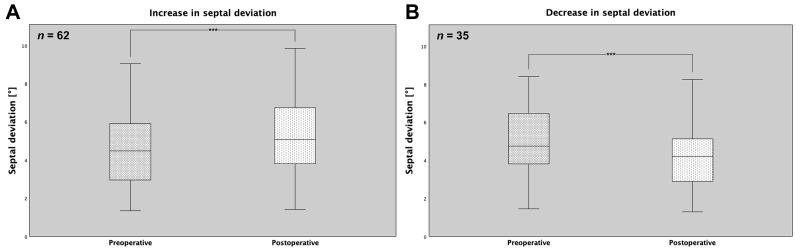
Pre- and postoperatively measured NSDs for increase (**A**) and decrease in septal deviation (**B**) with a highly statistically significant difference in each case (*** both *p* < 0.001).

Looking at the changes in NSD and dividing them into subgroups, an average deviation of 4.58° with a standard deviation of 2.03° (minimum 1.35° and maximum 10.5°) was found in the cases with an increase preoperatively and an average value of 5.28° with a standard deviation of 2.12° (minimum 1.4° and maximum 11.35°) postoperatively. In contrast, cases with a decrease in NSD showed an average value of 5.33° preoperatively with a standard deviation of 2.61° (minimum 1.45° and maximum 15.2°) and an average deviation of 4.47° postoperatively with a standard deviation of 2.36° (minimum 1.3° and maximum 14.7°). Here, the paired Wilcoxon signed-rank test revealed a statistically highly significant difference both between the pre- and postoperative NSDs in the subgroup with a terminal increase in the degree of deviation (*** *p* < 0.001, Figure 4A) and between the pre- and postoperative NSDs in cases with a final decrease (*** *p* < 0.001, Figure 4B). In the paired analysis, an increase in NSD was observed in a total of 62 patients, while a decrease in deviation was present in 35 cases. In summary, these data indicated that LF-IO could both worsen and improve an NSD. All the results are shown in Table 2.

### 3.3. Correlation of Changes in NSD with Surgery-Related Parameters

In a further step, an attempt was made to identify parameters that could have an effect on the increase or decrease in NSD. Age correlated positively with the increase in deviation (*r* = 0.28, *p* = 0.014, CI: −1.0–−0.068), whereas no significant correlation was found for the decrease in NSD (*r* = 0.051, *p* = 0.386, CI: −0.243–1.0). For gender, no significant correlation was observed for either an increase (*r* = −0.136, *p* = 0.146, CI: −1.0–0.083) or a decrease in NSD (*r* = −0.02, *p* = 0.454, CI: −1.0–0.272). When subgroups were formed on the basis of isolated maxillary movements, a significant correlation was observed for an anterior displacement of the maxilla with a decrease in NSD (*r* = 0.296, *p* = 0.042, CI: 0.006–1.0), but this direction of movement did not correlate significantly with the increase (*r* = 0.138, *p* = 0.142, CI: −0.081–1.0). The cranial repositioning of the maxilla had no influence on the increase (*r* = −0.054, *p* = 0.339, CI: −1.0–0.165) and decrease in NSD (*r* = 0.087, *p* = 0.309, CI: −0.209–1.0), as the correlation was not significant in each case. Similarly, the bivariate correlation analysis showed no significant correlation with the increase (*r* = −0.094, *p* = 0.237, CI: −1.0–0.128) and decrease in NSD (*r* = 0.039, *p* = 0.411, CI: −0.254–1.0) for a caudal movement of the maxilla. The *p*-values mentioned are listed together with the correlation coefficients in Table 3.

## 4. Discussion

In the present study, the occurrence of NSDs after LF-IO was investigated and an attempt was made to identify possible risk factors that have influence on a septal deviation. The necessary angle measurements of the nasal septum were performed using pre- and postoperative CBCT images in order to enable a precise examination due to their similarity to computer tomography (CT) images. On the one hand, a recommendation for the assessment of the sinonasal region using CBCT images had already been formulated in earlier studies [25]. On the other hand, Andrades et al. were able to demonstrate a similar accuracy of CT images compared to nasal endoscopy in their study [26].

Contrary to the results of Moroi et al. and Baeg et al., the results of this study showed a change in the nasal septum in 97 of 102 cases in postoperative examinations [19,20]. Highly significant differences were observed between pre- and postoperative CBCT images in form of an increase as well as a decrease in NSD (both *p* < 0.001). The influence of maxillary displacement on the expression of NSDs has already been addressed in previous studies and On et al. came to the conclusion that an impaction movement entails the risk of NSDs [17]. The reason for this was the need to shorten the nasal septum by the same amount by which the maxilla was moved cranially and an additional shortening of three millimeters to prevent the septum from hitting the nasal floor and thus deviating laterally [1,13,17,27]. The value of two millimeters of impaction was cited by On et al. as the threshold for an increased risk of an NSD, and they also identified a second risk factor for the occurrence of deviation with the anterior displacement of the maxilla, especially for the anterior region of the septum [17]. However, a positive correlation of the anterior displacement of the maxilla with a decrease in NSD was observed in the present study (*r* = 0.296, *p* = 0.042, CI: 0.006–1.0). Accordingly, the NSD decreased with greater anterior displacement of the maxilla. One explanation could be the fact that in this study the NSD was measured at the level of the transition from the cartilaginous to the bony area of the septum, in contrast to On et al. [17]. The surgical procedure itself could provide another possible explanation for this. The standard ostectomy of the cartilaginous and bony part of the septum in our clinic could result in little or no interference between the nasal septum and the nasal floor when the maxilla is moved forward. Furthermore, no influence of a cranial or caudal movement of the maxilla or of gender on the change in NSD could be found in this study.

In addition, and in contrast to the results of Asan et al. who found no influence of age on NSDs, a statistically significant correlation between age and the increase in NSD was observed in this study (*r* = 0.28, *p* = 0.014, CI: −1.0–−0.068) [1]. Thus, the deviation of the nasal septum increased with age and consequently younger patients showed less deviation. One possible reason for this observation could be the change in the cartilaginous structure of the septum with advancing age. The results of previous studies have shown that the cartilaginous part of the nasal septum becomes less stiff and weaker with age [28,29,30]. In this context, Kim et al. found a decrease in glycosaminoglycan in septal cartilage tissue [28]. These age-related structural changes can result in the nasal septum being less resistant, for example to the physical force exerted during preparation or shortening of the septum. Further preliminary work has also described nasal intubation, or more precisely the subsequent extubation, as a risk factor for the occurrence of an NSD [7,27]. If the cartilage of the septum has changed in its stiffness and hardness due to advanced age, as described above, the pressure of the tube during extubation can lead to greater deviation than in younger patients. In further studies, the possibility of submental intubation was mentioned as an alternative to the nasal variant [7,31]. This technique not only offers a good surgical field of vision but also avoids the risk of an NSD following extubation [7]. However, from an esthetic point of view, postoperative scarring is a disadvantage [31].

Nevertheless, this study has some limitations. Firstly, the retrospective approach and the manual determination of reference points for measuring the angle of the respective NSD must be mentioned here. This entails a certain susceptibility to error, which, however, was attempted to be minimized by the independent evaluation of two different investigators. In this regard, the use of computer-aided determination of measuring points and automated angle measurement could represent a feasible approach for future studies. The retrospective analysis of data using the aforementioned inclusion criteria entails the risk that only a subgroup of the population was included and that the results cannot be generalized as a result. In addition, the number of increases in NSD was not negligibly greater than the number of cases with a decrease in deviation, which proved to be particularly difficult when searching for potential risk factors. Thus, we attributed the non-significant result and the lack of a negative correlation of age with the decreases in NSD to the smaller sample size. However, further studies are needed to identify additional parameters that could influence NSDs. In a future prospective study, taking into account the results of this work, a certain age threshold could be identified above which the risk of an increase in NSD after LF-IO can rise significantly. Consequently, in future cases, the septum could be reduced more in the context of LF-IO, for example, in order to prevent NSDs and thus achieve a clinically better result [27]. Conversely, the intraoperative shortening of the septum could be performed more sparingly in younger patients below this risk age limit.

## 5. Conclusions

In summary, the results of this study show that LF-IO has a statistically significant influence on NSDs and can both exacerbate and attenuate it. Furthermore, it was observed that age has a significant influence on a postoperative increase in NSD. In this respect, it can be concluded that the risk of increasing deviation after LF-IO rises with age. In addition, it could be demonstrated that an appropriate ostectomy of the posterior bony septum reduces the risk of septal deviation after maxillary advancement.

## Figures and Tables

**Table 1:** Clinicopathological characteristics of the patient cohort.

Category	Total (*n* = 102)
Gender:	
Female	60 (58.8%)
Male	42 (41.2%)
Age (MV in years)	28.12 (17 to 56)
Diagnosis:	
Class II Dysgnathia	50 (49%)
Class III Dysgnathia	47 (46%)
Circular open bite	2 (2%)
Front open bite	1 (1%)
Mandibular retrognathism	1 (1%)
Mandibular prognathism	1 (1%)
Maxillary movement:	
Cranial	36
Anterior	96
Caudal	18
Classification by Mladina:	
Class 0	4 (3.9%)
Class 1	3 (2.9%)
Class 2	11 (10.8%)
Class 3	19 (18.6%)
Class 4	12 (11.8%)
Class 5	29 (28.4%)
Class 6	13 (12.8%)
Class 7	11 (10.8%)
Increase in NSD:	
Grade I	33 (32.3%)
Grade II	27 (26.5%)
Grade III	2 (2%)
Decrease in NSD:	
Grade I	18 (17.6%)
Grade II	15 (14.7%)
Grade III	2 (2%)
Unchanged septum	5 (4.9%)

MV = mean value; NSD = nasal septal deviation.

**Table 2:** Pre- and postoperative changes in NSD.

(*n* = 62)	Increase in NSD (°)		(*n* = 35)	Decrease in NSD (°)	
	Preoperative	Postoperative		Preoperative	Postoperative
Mean	4.58	5.28		5.33	4.47
Median	4.48	5.08		4.75	4.2
SD	2.03	2.12		2.61	2.36
Minimum	1.35	1.4		1.45	1.3
Maximum	10.5	11.35		15.2	14.7
*p*-value	<0.001			<0.001	

NSD = nasal septal deviation; SD = standard deviation.

**Table 3:** Correlation of changes in NSD with indicated parameters. Values represent Spearman correlation coefficients.

	Changes in NSD			
Category	Increase (*n* = 62)	CI	Decrease (*n* = 35)	CI
Age	0.28 *p* = 0.014	−1.0–−0.068	0.051 *p* = 0.386	−0.243–1.0
Gender	−0.136 *p* = 0.146	−1.0–0.083	−0.02 *p* = 0.454	−1.0–0.272
Cranial movement	−0.054 *p* = 0.339	−1.0–0.165	0.087 *p* = 0.309	−0.209–1.0
Anterior movement	0.138 *p* = 0.142	−0.081–1.0	0.296 *p* = 0.042	0.006–1.0
Caudal movement	−0.094 *p* = 0.237	−1.0–0.128	0.039 *p* = 0.411	−0.254–1.0

NSD = nasal septal deviation; CI = confidence intervals.

## Data Availability

Data can be obtained upon request by scientists who are conducting work independent of commercial interests. Data are not stored on publicly available servers.
